# Giant paratesticular liposarcoma with lung metastases: a case report and review of the literature

**DOI:** 10.1186/s13256-020-02420-x

**Published:** 2020-07-02

**Authors:** Takuro Noguchi, Toshirou Fukushima, Hiroaki Hara, Nodoka Sekiguchi, Takashi Kobayashi, Takesumi Ozawa, Daisuke Gomi, Tomonobu Koizumi

**Affiliations:** 1 Department of Comprehensive Cancer Therapy, Shinshu University School of Medicine, 3-1-1 Asahi, Matsumoto, 390-8621 Japan; 2 Department of Urology, Shinshu University School of Medicine, 3-1-1 Asahi, Matsumoto, 390-8621 Japan

**Keywords:** Paratesticular liposarcoma, Chemotherapy, Pulmonary metastasis

## Abstract

**Background:**

Due to its rarity, little is known about the clinical presentations and responses to systemic chemotherapies in advanced and/or metastatic cases of paratesticular liposarcoma.

**Case presentation:**

Here, we report the case of a 75-year-old Japanese man with giant paratesticular liposarcoma. Imaging studies revealed a 26 cm tumor in his right scrotum and lung metastases at presentation. He underwent radical orchiectomy followed by systemic chemotherapies. Pathological findings of the resected primary tumor confirmed a dedifferentiated liposarcoma. He then started chemotherapy treatment with gemcitabine plus docetaxel. His disease status was stable for 1 year. Eribulin was used for second-line chemotherapy. He had a relapse at 5 months after eliburin and died at 22 months after diagnosis.

**Conclusion:**

Early diagnosis and curative radical surgery are important for treatment of paratesticular liposarcoma. However, a giant paratesticular liposarcoma could cause metastases, and systemic chemotherapy may be helpful for prolonging survival in patients with metastatic paratesticular liposarcoma.

## Background

Urological sarcomas are rare tumors accounting for 2.7% of all soft tissue sarcomas across organs in adult patients [1]. Paratesticular liposarcoma (PTL) is relatively common among the urological sarcomas in the literature [1–5], however, PTL is an extremely rare clinical entity. Based on previous case studies and recent reviews [3–9], approximately more than 200 cases of PTL have been reported to date. PTLs mostly present as a painless, slow-growing inguinal or inguinoscrotal mass [2, 3, 8, 9]. In general, surgical approaches, including radical orchiectomy with wide local excision and high ligation of the spermatic cord, should be performed in cases of suspected PTL [2–5]. However, 1–5% of patients with paratesticular sarcoma, including PTL, present with metastatic disease [2–5]. Their extremely low incidence hampers our understanding of metastatic PTLs in terms of clinical presentations, effective chemotherapeutic regimens, and prognosis. We encountered a case of a patient with giant PTL with lung metastases at the initial diagnosis. Here, we report its clinical course because the present case had a huge paratesticular liposarcoma, and relatively prolonged control was obtained by serial chemotherapies. We also present a review of the disease and discuss the role of chemotherapies.

## Case presentation

**Fig. 1:**
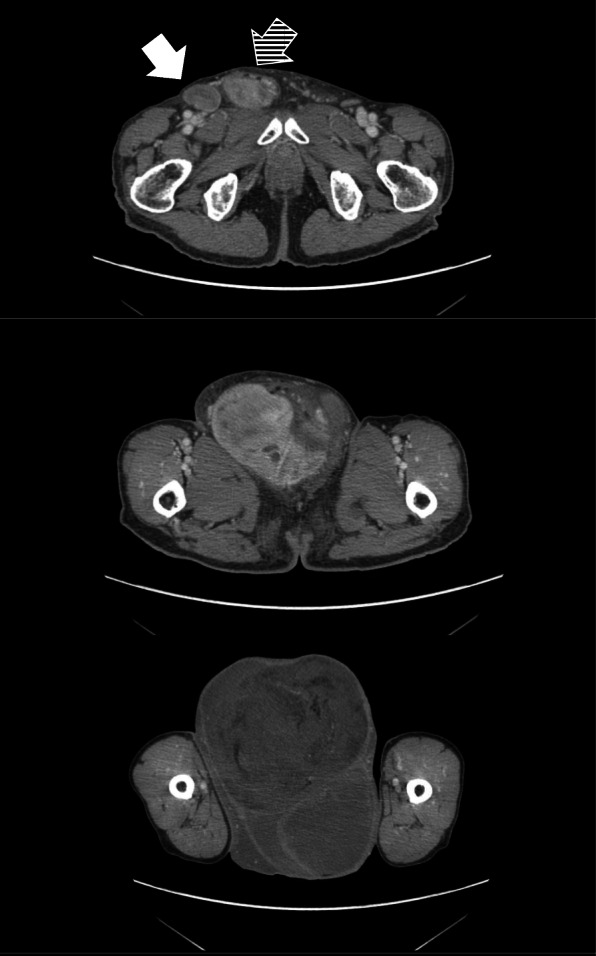
Enhanced computed tomography scan revealed a solid tumor in the right scrotum. The tumor consisted of the enhanced lesion (*middle*) and one with adipose density (*bottom*). Right inguinal lymphadenopathy (*top, white arrow*) and the extended tumor in the inguinal canal (*top, striped arrow*) were observed

**Fig. 2:**
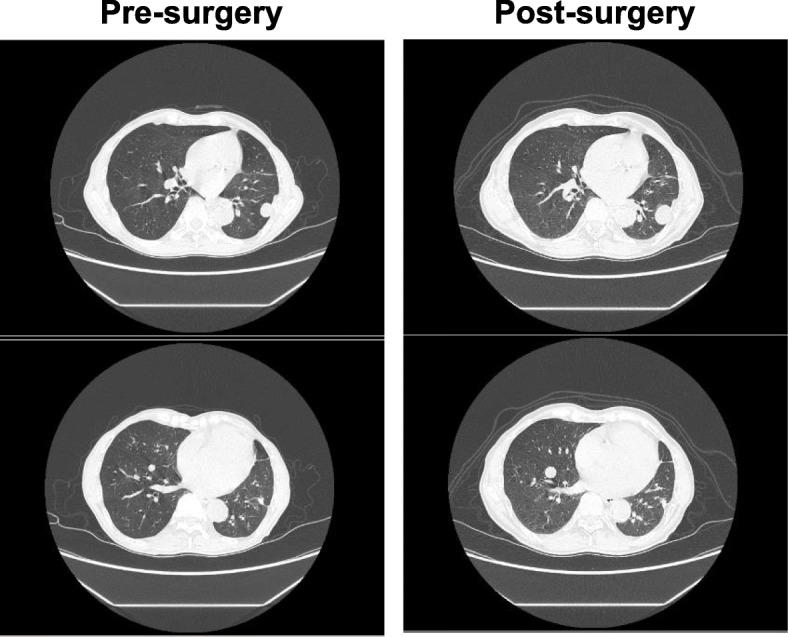
Multiple lung nodules were found at presentation (*left*). At 2 months after orchiectomy, the nodules were enlarged in size (*right*)

**Fig. 3:**
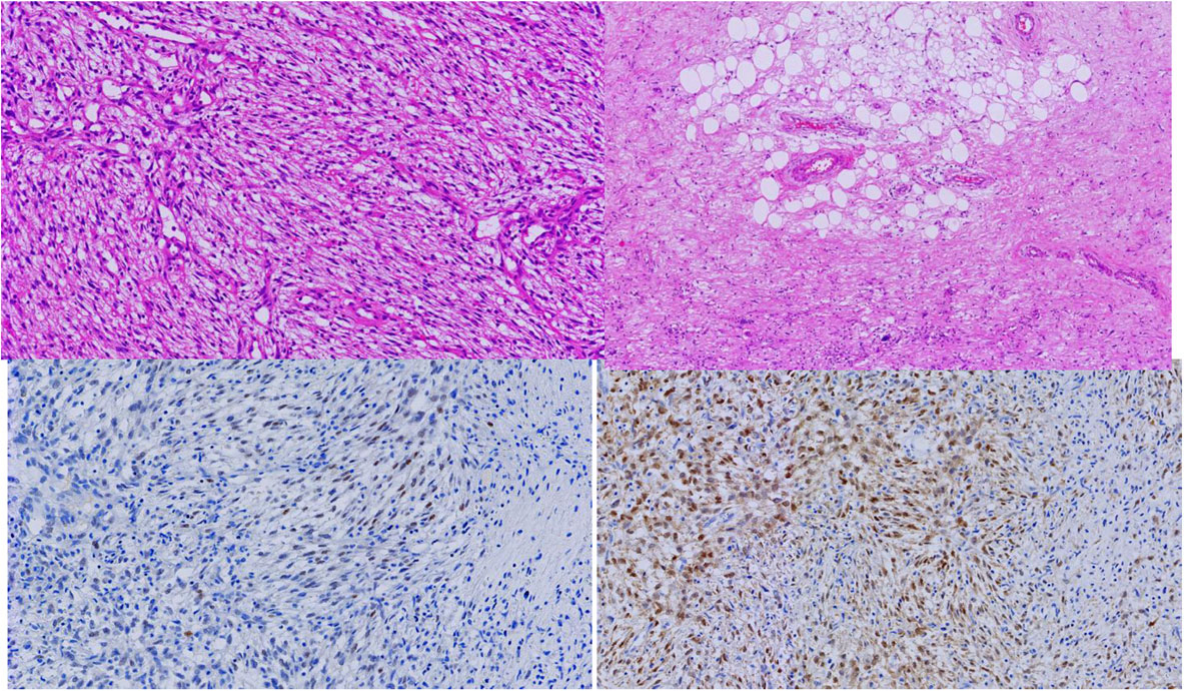
Histological findings revealed the proliferation of spindle and pleomorphic cells containing bizarre hyperchromatic nuclear cells with both poorly (*left upper*) and well-differentiated areas (*right upper*). They were positive for CDK4 (*left lower*) and MDM2 (*right lower*). Magnification, × 20

**Fig. 4:**
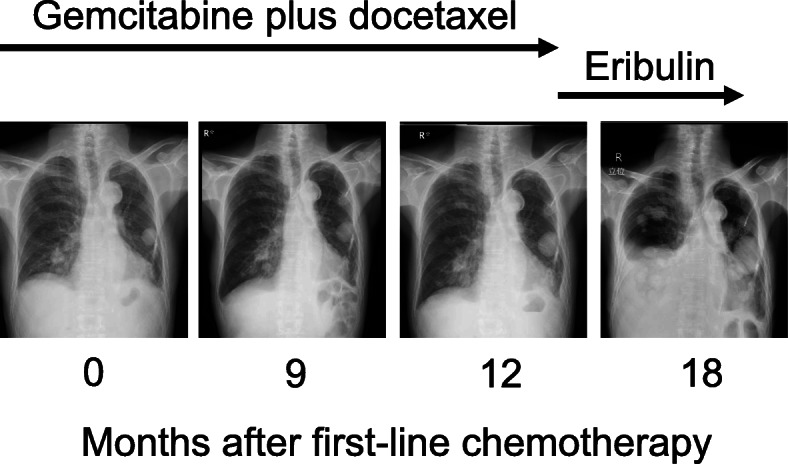
Serial chest radiographic findings after chemotherapy

A 75-year-old Japanese man complained of a swollen right scrotum, which had grown slowly for 1 year. A huge elastic hard mass was palpable in his right scrotum. The surface of the skin showed no redness, rash, ulcers, or other skin abnormalities. He had neither pain nor obstruction of urination. He had worked as a farmer for a long time. He had a smoking history (20 pack-year) but did not consume alcohol. There was no history of disease or surgery. There was no specific family history, including of cancer. Physical and neurological examinations were normal, except for a 26 cm tumor in his right scrotum and right inguinal lymphadenopathy (3 cm). His blood pressure was 146/80, heart rate 86, and temperature 36.8 degrees. Laboratory findings are shown in Table 1; there were no abnormal findings. The findings by computed tomography (CT) were shown in Fig. 1a. In addition, multiple lung nodules were detected on chest CT (Fig. 2). Our patient underwent radical orchiectomy and lymph node dissection. Histological analysis revealed the proliferation of spindle and pleomorphic cells containing bizarre hyperchromatic nuclear cells with both poorly and well-differentiated areas. On immunohistochemistry, tumor cells were negative for cytokeratin AE1/AE3, desmin, S-100, STAT6, BCL-2, and MIC2, but positive for smooth muscle actin, CDK4, and MDM2 (Fig. 3). These findings led to a pathological diagnosis of dedifferentiated PTL. Histological examination showed no microscopic invasion to the testis. Based on the soft tissue sarcoma classification, the disease was T2bN1M1 (IV) stage. At 2 months after surgery, the lung nodules showed rapid progression (+ 40% for 2 months) (Fig. 2). First-line treatment was then started with gemcitabine (1000 mg/m^2^ day 1) plus docetaxel (30 mg/m^2^, day 1) chemotherapy every 2 weeks. The metastasis in his left lung was set as a target lesion by chest radiography. The time course of the imaging studies is shown in Fig. 4. The disease was well controlled by gemcitabine plus docetaxel chemotherapy for 1 year without any adverse events until its progression. We subsequently performed second-line eribulin (1.4 mg/m^2^, day 1, 8, every 3 weeks) chemotherapy. Despite the progression of the lung metastases, his symptoms remained stable. However, at 4.5 months after second-line chemotherapy, chest radiography showed progression of metastatic nodules and the presence of pleural effusion. In all, he underwent chemotherapy for 18 months and died at 22 months after diagnosis. An autopsy was not performed.

**Table 1:**
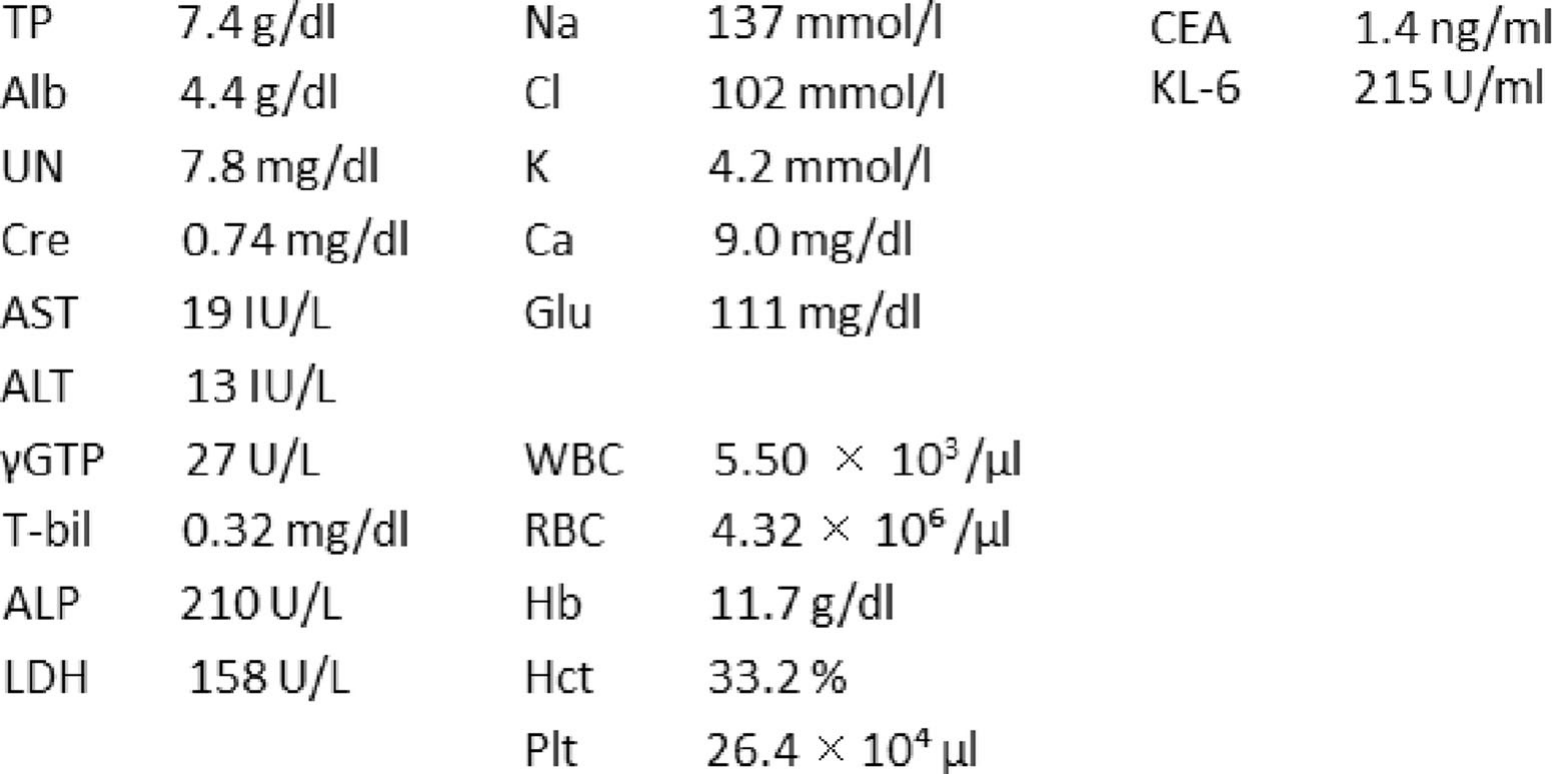
Blood chemistroies on admission

## Discussion

We described a case of a patient with giant paratesticular liposarcoma initially presenting with pulmonary metastases, and the treatment outcome along with serial chemotherapies. Such a huge tumor is extremely rare, and little information about the usefulness of chemotherapy exists about paratesticular liposarcoma.

Malignant liposarcoma is classified into the following subtypes: dedifferentiated, myxoid/round cell, and pleomorphic liposarcoma [10]. Like other liposarcomas, the prognosis of PTL depends on its histology. The well-differentiated (intermediate of lipomatous tumors) and myxoid/round cell types have a better prognosis, but they tend to have high incidence rates of local recurrence [2, 3, 11]. PTL typically affects adults, with the most common age at presentation being 50–60 years [5]. Diagnosis of PTL is challenging in some cases because their clinical manifestations mimic groin hernia, hydrocele, or other benign lesions [12, 13]. Testicular tumors more than 10 cm in diameter are called “giant” [13]. Several giant PTLs have been reported [8, 12, 14]. However, the size of 26 cm in our case was relatively huge even among these previous reports.

There were no metastatic lesions in organs other than lungs, and systemic chemotherapy was initiated in the present case. The response rates of chemotherapies, such as doxorubicin, ifosfamide alone, or in combination, are generally low and the response rates to these agents also vary according to histological types in soft tissue sarcomas [15]. Jones *et al.* examined the differential sensitivity of liposarcoma subtypes to chemotherapy [16]. Myxoid liposarcoma was relatively chemo-sensitive compared to dedifferentiated or well-differentiated liposarcoma. Indeed, the response rate of dedifferentiated liposarcoma to first-line chemotherapy was only 25% (doxorubicin, 8%; doxorubicin plus ifosfamide, 17%). In our case, because of his advanced age and performance status, our patient was considered contraindicated for anthracycline-based chemotherapies, leading to the choice of gemcitabine plus docetaxel. In real-world data based on the Surveillance, Epidemiology and End Results (SEER) in the United States [17] and the medical record review in Europe [18], gemcitabine plus docetaxel chemotherapy was most commonly used in older patients with soft tissue sarcoma. In a phase 2, open-label study, this drug combination showed antitumor activities (response rate 16%) for metastatic soft tissue sarcomas, including liposarcoma [19]. Although complete response to gemcitabine plus docetaxel in a patient with retroperitoneal liposarcoma was reported [20], information about chemotherapeutic responses in PTL is limited. First-line chemotherapy using the combination of gemcitabine plus docetaxel continued for 1 year until disease progression in the present case. As the lung metastases progressed rapidly without systemic treatment after surgery for only 2 months, the chemotherapy used here was conceivably efficacious.

Eribulin, a microtubule growth inhibitor, was highly efficacious in patients with advanced liposarcoma [21]. However, second-line chemotherapy using eribulin was unlikely to be effective in the present case, because of early progression of pulmonary metastatic lesions.

In the SEER data, median survival time in registered patients with advanced liposarcomas was 21.1 months [17]. The survival time of 22 months in our case initially presenting giant primary tumor and lung metastasis was comparable to other reports. Therefore, although evidence on effective chemotherapies for PTL is limited, we believe that systemic chemotherapy could have contributed to the survival in the present case. Dedifferentiated liposarcomas have genetic abnormalities with high-level amplification of chromosome 12q14–15, which includes the MDM2 and CDK4 cell cycle oncogenes [11, 22]. Based on the phase 2 trial of the CDK4 inhibitor, palbociclib [22], there was one complete response among 59 evaluable patients and the median progression free survival (PFS) was 17.9 weeks (two-sided 95% CI: 11.9–24.0 weeks). Further investigations of the molecular characteristics would be useful for the development of novel therapeutic targeted agents or to understand the etiology and risk factors in patients with PTL.

## Conclusion

We described a case of giant paratesticular liposarcoma initially presenting with pulmonary metastases, along with the treatment outcome. Although systemic chemotherapy may be helpful for prolonging survival, further studies regarding therapeutic strategies for inoperable and metastatic paratesticular liposarcomas are warranted.

## Data Availability

Not applicable.
